# Discovery of Natural Phosphodiesterase 5 Inhibitors from *Dalbergia cochinchinensis* Pierre Leaves Using LC-QTOF-MS^2^

**DOI:** 10.3390/plants14111652

**Published:** 2025-05-29

**Authors:** Ruttanaporn Chantakul, Corine Girard, François Senejoux, Kornkanok Ingkaninan, Nitra Nuengchamnong, Prapapan Temkitthawon

**Affiliations:** 1 Center of Excellence for Natural Health Product Innovation and Center of Excellence for Innovation in Chemistry, Faculty of Pharmaceutical Sciences, Naresuan University, Phitsanulok 65000, Thailand; ruttanapornc62@nu.ac.th (R.C.); k_ingkaninan@yahoo.com (K.I.); 2 Université Marie et Louis Pasteur, EFS, INSERM, UMR RIGHT, F-25000 Besançon, France; corine.girard@univ-fcomte.fr (C.G.); francois.senejoux@univ-fcomte.fr (F.S.); 3 Science Laboratory Centre, Faculty of Science, Naresuan University, Phitsanulok 65000, Thailand; nitra_nuengchamnong@yahoo.com

**Keywords:** rosewood, at-line LC-QTOF-MS^2^, erectile dysfunction, phosphodiesterase 5

## Abstract

The imbalance of phosphodiesterase 5 (PDE5) enzyme in the male body, or excessive PDE5 enzyme levels, can occur due to factors such as aging, diseases (e.g., cardiovascular disease, diabetes, depressive disorder), and physical behaviors (e.g., alcoholism, smoking, stress). PDE5 is directly associated with erectile dysfunction disease. Currently, many studies aim to find natural PDE5 inhibitors as an alternative to commercial drugs. This study is the first to demonstrate that the ethanolic leaf extract of *D. cochinchinensis* exhibits potent PDE5-inhibitory activity. The PDE5-inhibitory activity of five plant parts was evaluated: leaf (IC_50_ = 1.53 ± 0.12 µg/mL), twig (3.37 ± 0.54), fruit (14.92 ± 2.85), heartwood (19.05 ± 5.60), and bark (16.03 ± 2.92). However, there is still uncertainty about which compounds in leaf extract are responsible for the PDE5 inhibition. Therefore, the purpose of this study is to identify the chemical constituents in the leaf of *D. cochinchinensis*, including determining which of these compounds may act as PDE5 inhibitors. This study was achieved using at-line LC-QTOF-MS^2^.

## 1. Introduction

Erectile dysfunction is a prevalent male sexual disorder characterized by the inability to achieve or maintain an erection sufficient for satisfactory sexual performance [1,2]. The risk factors contributing to ED include aging, lifestyle choices, medications, chronic diseases, pelvic injuries, and abnormalities in endocrine and cardiovascular systems [3]. ED involves cyclic contraction and the relaxation of corpus cavernosum smooth muscle, which is regulated by phosphodiesterase 5 (PDE5)—mediated cGMP hydrolysis [1,4,5,6]. PDE5 is a cyclic guanosine monophosphate (cGMP)-specific enzyme predominantly found in the lungs, smooth muscles, platelets, and corpus cavernosum [4,5]. It hydrolyzes cGMP to its inactive form, guanosine monophosphate (GMP), leading to decreased cGMP levels and vascular smooth muscle contraction. Conversely, inhibiting PDE5 promotes smooth muscle relaxation, which is essential for penile erection [7]. Although FDA-approved PDE5 inhibitors (e.g., sildenafil, tadalafil) effectively restore erection, adverse effects (e.g., headache, flushing) and contraindications (e.g., nitrate co-administration) limit their use [8,9,10].

Research interest in natural products has been steadily growing as scientists aim to discover safer and more affordable PDE5 inhibitors [11]. Flavonoids in medicinal plants have been reported as potential sources of PDE5 inhibitors [12,13,14]. In addition, curcuminoids [15], xanthones [16], phenanthrenes [17], and alkaloids [18] have been reported to exhibit PDE5 inhibitory activity. Several species of the *Dalbergia* genus are traditionally used in various medicinal systems and have been reported to exhibit a wide range of bioactivities, such as anti-inflammatory, osteogenic, antioxidant, anti-androgenic, antibacterial, anti-allergic, antidiarrheal, antifungal, anti-tumor, and blood stasis [19,20]. Phytoconstituents isolated from heartwood, including stem and seeds of *D. cochinchinensis*, have been reported to include flavonoids (flavones, isoflavones, flavans, isoflavans, flavanones, chalcones, neoflavonoids, and pterocarpans) and simple phenols as major compounds, along with terpenoids (sesquiterpenoids and triterpenoids), quinones, benzofurans, benzophenols, phytosterols, stilbenes, xanthones, and lignans [21,22,23,24]. The *D. cochinchinensis* seed is rich in glycosidase enzymes, with both β-glucosidase and β-fucosidase activities [25]. From reviews, chemical compounds in the leaf have been reported in only four species of *Dalbergia*, namely *D. sissoo*, where flavonoids (including flavones and isoflavones), triterpenoids, and phytosterols have been identified [26,27,28,29], *D. stipulacea*, which has been found to contain luteolin, *D. spruceana*, which has been reported to contain luteolin 4′-rutinoside, and *D. spinosa*, which has been noted to contain prunasin [30,31]. *D. cochinchinensis* has demonstrated its various bioactivities, including antioxidant [19], antimicrobial effects against Staphylococcus aureus and Aspergillus niger, cytotoxicity against lung and pericardial cancers [32], 5α-reductase inhibition [33], and anti-inflammation effects [34].

Using a chemotaxonomic approach to guide the selection of plants for PDE5 inhibition screening, *D. cochinchinensis* was chosen based on reports of its chemical composition, particularly its flavonoid content—compounds previously reported to exhibit PDE5 inhibitory activity. Additionally, ethnopharmacological records indicate the traditional use of *D. cochinchinensis*, especially its heartwood, for treating tumors and blood stasis [20]. Furthermore, based on our literature review, no previous studies have reported the PDE5 inhibitory activity of this plant. These findings collectively supported the selection of this species for further investigation. To the best of our knowledge, no previous studies have reported the PDE5 inhibitory activity of *D*. *cochinchinensis*. This study is the first to demonstrate such activity, providing new insights into the pharmacological potential of this species. In preliminary screening, we found that the hydro-ethanolic leaf extract inhibited PDE5 most potently, motivating detailed LC-QTOF-MS^2^ profiling.

## 2. Results

### 2.1. PDE5 Inhibitory Activity of Various Parts of D. cochinchinensis

In our preliminary study, five parts of *D. cochinchinensis* (leaf, twig, fruit, heartwood, and bark) were extracted with 95% ethanol and screened for PDE5 inhibitory activity. Table 1 shows that all extracts exhibited over 70% inhibition at a concentration of 25 µg/mL. Extracts exceeding 70% inhibition were considered effective and selected for IC_50_ determination. Among them, the leaf extract demonstrated the highest potency and was chosen for further identification of PDE5 inhibitors.

### 2.2. At-Line Screening and Identification of PDE5 Inhibitors in D. cochinchinensis Leaf Ethanolic Extract

**Figure 1:**
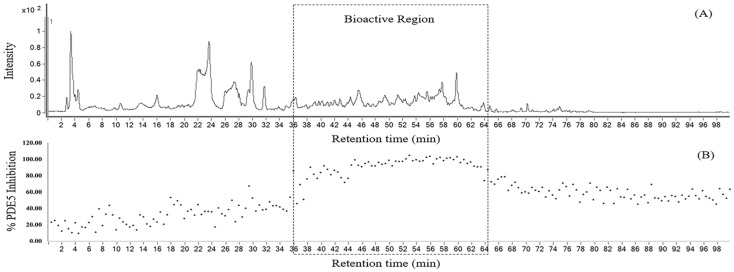
Extracted ion chromatogram from at-line LC-QTOF-MS of *D. cochinchinensis* leaf extract (**A**), and the corresponding bioactivity chromatogram representing the PDE5 inhibitory activity of collected micro-fractions (**B**).

**Figure 2:**
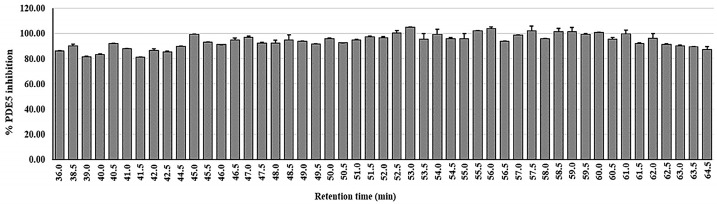
Graphical presentation of PDE5 inhibitory activities for at-line micro-fractions of *D. cochinchinensis* leaf ethanolic extract, with data expressed as mean ± standard deviation (*n = 3*).

The leaf ethanolic extract underwent rapid screening to identify the chemical compounds in the leaf of *D. cochinchinensis* using at-line LC-QTOF-MS^2^. Figure 1 displays the extracted ion chromatogram of the leaf extract in panel A, and the corresponding bioactivity chromatogram representing the PDE5 inhibitory activity of collected micro-fractions is displayed in panel B. The zoom-in of this chromatogram highlights the bioactive region, between 36 and 64 min of retention time. In this area, the micro-fractions demonstrated PDE5 inhibition of 80% or greater, as shown in Figure 2.

### 2.3. Identification of Chemical Compounds in the Leaf of D. cochinchinensis Using LC-QTOF-MS^2^

For the tentative identification of constituents of the leaf extract, the micro-fractions were analyzed using *m*/*z* fragmentation and MassHunter Data Acquisition Software Version B.05.01 and MassHunter Qualatative Analysis Software B 06.0 respectively (Agilent Technologies, Santa Clara, CA, USA). The chemical compounds in the leaf extract were identified based on *m*/*z* fragmentation and are listed in Table 2. From previous studies, a summary of compounds isolated from the leaves of various *Dalbergia* species is shown in Table 3.

### 2.4. Liquid–Liquid Partition of D. cochinchinensis Leaf Extract

**Scheme 1:**
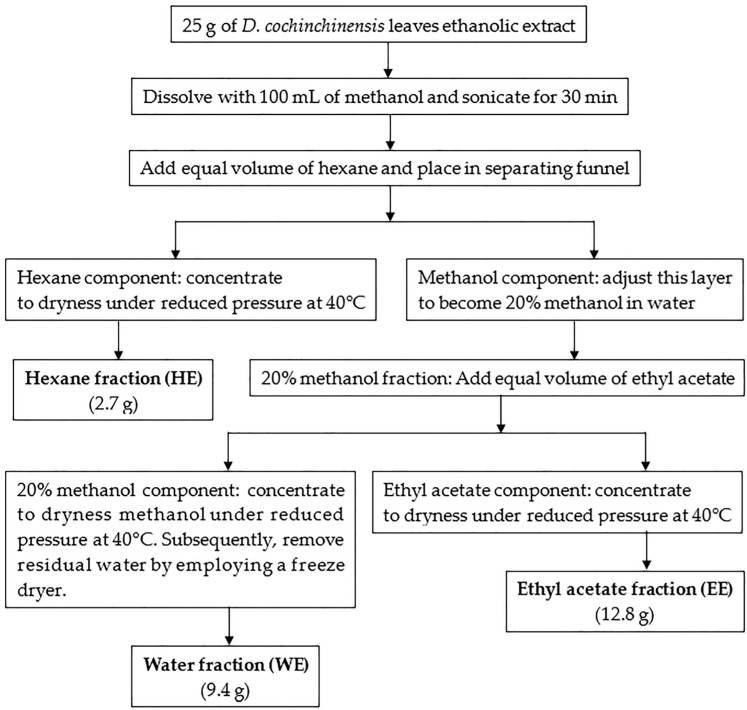
The flow chart indicates the sequential partitioning of *D. cochinchinensis* leaf ethanolic extracts.

**Figure 3:**
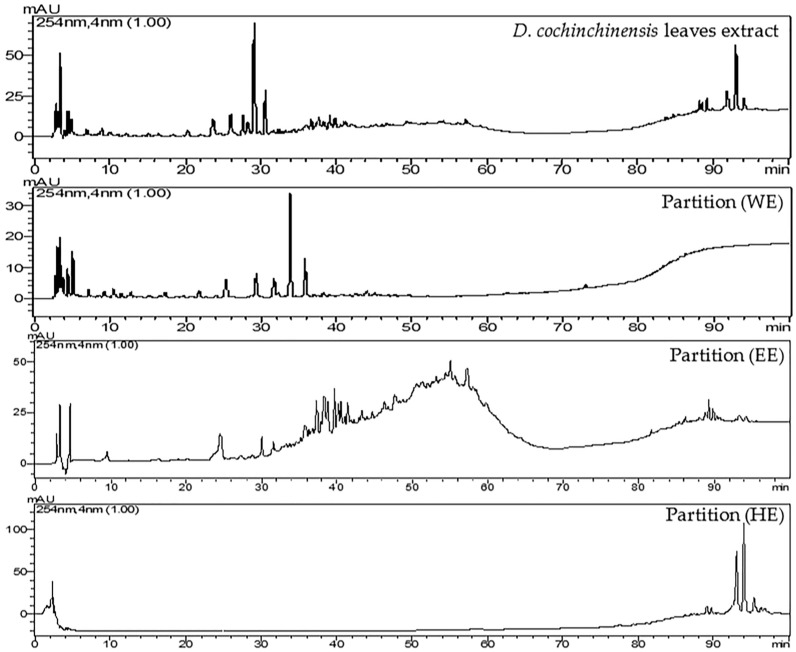
HPLC chromatograms of *D. cochinchinensis* leaf ethanolic extract and three fractions from partition with solvents: water (WE), ethyl acetate (EE), and hexane (HE) at 254 nm.

The study initially conducted at-line screening to assess both the biological activity and chemical composition in the leaf extract of *D. cochinchinensis*. The results revealed the chemical composition of the leaf extract and identified a group of substances expected to be the PDE5 inhibitor. Liquid–liquid partitioning was used to separate groups of compounds in the leaf extract based on the polarity of the solvents used. *D. cochinchinensis* leaf extract was fractionated using the partition method to segregate it into groups based on the polarity of the solvent. For sequential partitioning, 25 g of the extract were utilized. Liquid–liquid partition produced an extract of hexane (HE), ethyl acetate (EE), and water (WE) at 2.7 g, 12.8 g, and 9.4 g, respectively. The process of partition and the dry weight of samples obtained are stated in Scheme 1. Subsequently, these extracts were analyzed using HPLC, with the system conditions set to match those used for the at-line technique. The HPLC chromatograms of each extract are shown in Figure 3.

**Figure 4:**
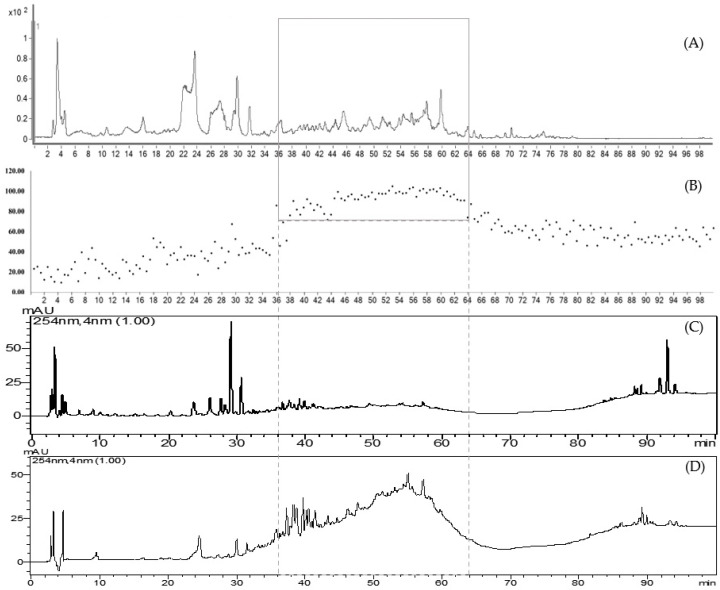
Comparison of the bioactive regions in the at-line technique (**A**,**B**), the HPLC chromatogram showing the chemical profile of the leaf extract (**C**), and the compounds in the EE partition extract (**D**) from *D. cochinchinensis*.

HPLC chromatograms of the partitioned extracts were compared with the crude extract. The chromatograms show that the extracts obtained from partitioning with different solvents clearly separate into distinct groups, including highly polar compounds (represented by the water extract, WE), moderately polar compounds (represented by the ethyl acetate extract, EE), and non-polar compounds (represented by the hexane extract, HE). All three partition extracts were tested for PDE5 inhibitory activity in triplicate at the final concentration of 5 μg/mL. The results revealed that the EE extract (95.69 ± 2.97%) demonstrated the highest efficacy in inhibiting PDE5, followed by the HE (65.57 ± 3.13%) and WE extracts (39.61 ± 2.75%), respectively. Upon examining the HPLC chromatogram of the EE extract, it was observed that it corresponds to the bioactive region identified in the at-line analysis, which contains compounds with more than 80% efficacy in inhibiting PDE5 (Figure 4). The study results confirm that the PDE5 inhibitors are indeed located in the bioactive region, and this also supports the validity of the at-line analysis technique.

### 2.5. Identification of Chemical Compounds of Ethyl Acetate Partition (EE) Using LC-QTOF-MS^2^

**Figure 5:**
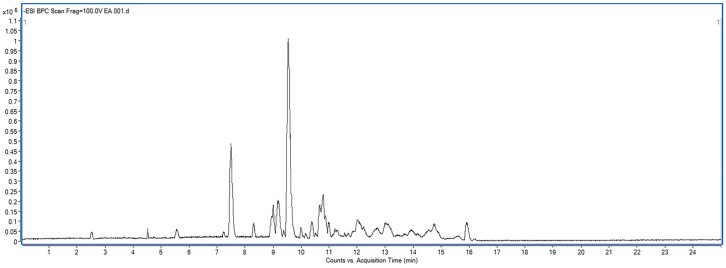
Extracted ion chromatogram from at-line LC-QTOF-MS of EE partition (negative mode).

The results indicate that the EE extract contains compounds with potential PDE5 inhibitory activity. Therefore, the EE extract was analyzed using LC-MS/MS to identify the tentative compounds based on *m*/*z* fragmentation patterns. The extracted ion chromatogram from at-line LC-QTOF-MS of EE partition (negative mode) is shown in Figure 5. The tentative identifications based on *m*/*z* fragmentation of the EE partition from *D. cochinchinensis* are listed in Table 4.

## 3. Discussion

*Dalbergia* genus includes 274 accepted species worldwide, with 26 native species found in Thailand [32]. *D. cochinchinensis*, traditionally known as Thai Rosewood or Siamese Rosewood, was described in 1898. It belongs to the family of Fabaceae and is widely distributed across Southeast Asia, including Vietnam, Thailand, Laos, and Cambodia. *D. cochinchinensis* is a large tree known for its durable heartwood, which is resistant to termites. This high economic value makes it sought after for various commercial purposes, including seeds, living trees, heartwood, handicrafts, and furniture [36].

The screening of PDE5 inhibitory activity in *D. cochinchinensis* revealed that the leaf extract exhibited the lowest IC_50_ values, indicating the highest efficacy, followed by the twig, fruit, bark, and heartwood, respectively. This study demonstrates, for the first time, that the leaf of *D. cochinchinensis* possesses the most potent PDE5 inhibitory activity. Therefore, it was of interest to further investigate the active compounds in the leaf, as no reports have yet identified chemical compounds from *D. cochinchinensis* acting as PDE5 inhibitors.

The at-line LC-QTOF-MS^2^ technique was used for rapid screening of bioactivity coupled with mass fragmentation. The use of at-line analysis allowed for the identification of potential PDE5 inhibitory zones in the chromatogram of the leaf extract. However, it was not possible to isolate pure compounds from the collected micro-fractions, with the amount of the fractions remaining after evaporation being insufficient for further study. Thus, the use of at-line techniques serves as a rapid screening method, providing both biological activity assessment and predictive analysis of potential PDE5 inhibitors based on *m*/*z* data. This study presents a faster approach for lead identification and the dereplication of known compounds compared to conventional fractionation techniques. However, despite the efficiency of at-line analysis, there is a concern that compounds present in low natural abundance may be overlooked.

This study found that the leaf predominantly contains flavonoids, a class of compounds known for their significant biological properties. Additionally, other substances have also been found, including sugar group, phenyl propanoic acid, quinic acid, benzodioxoles, sesquiterpene, xanthone, hydroxycinnamic acid, proanthocyanidin, quinoline alkaloid, stilbene, chalcone, and lipid. The compounds found in the bioactive region include flavonoids such as kaempferol and quercetin glycosides, as well as other substances like guibourtinidol-(4α→6)-catechin, niaziminin, and liquiritigenin. Previous reports have isolated flavonoid glucosides, including kaempferol-3-O-β-D-glucopyranoside, kaempferol-3-O-rutinoside, quercetin-3-β-D-glucopyranoside, and quercetin-3-O-rutinoside from the leaf of *D. sissoo*, as well as liquiritigenin from the heartwood of *D. cochinchinensis* [21,37]. Literature reviews indicate that the compounds isolated from the leaf are predominantly flavonoids (including flavanones, flavones, and isoflavones), along with other compounds such as triterpenoids, phytosterols, and lignans [19,24]. The results from the at-line technique have revealed the presence of several of these compounds in the leaf of *D. cochinchinensis*. However, these data are preliminary, based on mass spectrometry, and provide an initial analysis of the compound groups. The results of our experiment can be used to hypothesize potential PDE5 inhibitory compounds (in the bioactive region) and serve as a basis for further isolation of pure substances from the leaf extract.

The ethanolic leaf extract was further subjected to sequential partitioning to isolate the bioactive fraction. The partitioning process revealed that the bioactive fraction was obtained using the ethyl acetate (EE) partition. The compounds predominantly found in the EE partition include flavonol glycosides and procyanidins. In addition, other substances were also found, such as polyphenol, *O*-glycosyl, phenolic acid, monocarboxylic acid, phenylpropanoid, phenolic aldehyde, procyanidin, cyanogenic glycoside, and alkaloid. The partitioning process enabled the separation of compound groups, allowing us to identify those with PDE5 inhibitory activity. This helped specify the active groups more precisely and guided the selection of fractions for further isolation in a more targeted manner. This study has led to the discovery of candidate compounds for PDE5 inhibitors in *D. cochinchinensis*, which is being reported for the first time. Especially, the flavonol glycosides include kaempferol and quercetin glycosides, along with procyanidins, which consist of catechin-afzelechin, afzelechin dimers and trimers, and afzelechin-guibourtinidol. These compounds may have promise in becoming a PDE5 inhibitor. This research provides information about compounds in the leaves of *D. cochinchinensis* that have the potential to act as PDE5 inhibitors. Nevertheless, further studies are needed to identify the active ingredients and to complete the research in the future.

## 4. Materials and Methods

### 4.1. Chemicals and Materials

#### 4.1.1. Plant Material

*D. cochinchinensis* was sourced from the Phra Mae Ya Sukhothai Botanical Garden in Sukhothai Province, Thailand. The voucher specimens (No. 004549) were authenticated by Assistant Professor Dr. Pranee Nangngam, Department of Biology, Faculty of Sciences, Naresuan University, Phitsanulok Province, Thailand.

#### 4.1.2. Solvents

The following chemicals were purchased from RCI Labscan (Bangkok, Thailand): 95% ethanol, ethyl acetate, hexane, acetonitrile, water, dimethyl sulfoxide (DMSO), 98% formic acid (analytical grade), acetonitrile (HPLC grade), and methanol (LC-MS grade).

#### 4.1.3. Reagents

Bovine serum albumin (BSA), crude snake venom (Crotalus atrox), dulbecco’s modified eagle medium (DMEM), dithiothreitol (DTT), ethylenediamine tetraacetic acid (EDTA), ethylene glycol tetraacetic acid (EGTA), fetal bovine serum (FBS), imidazole, magnesium chloride (MgCl_2_), phenylmethylsulfonyl fluoride (PMSF), QAE Sephadex™ A-25, and tris (hydroxymethyl) aminomethane (Tris) were purchased from Sigma–Aldrich (St. Louis, MO, USA). Geneticin (G418) was purchased from Gibco by Life Technologies (Paisley, Scotland). [^3^H]-cGMP and Ultima Gold cocktail were obtained from PerkinElmer (Waltham, MA, USA).

#### 4.1.4. Tools Used for Analysis

ESI mass spectra were determined using an Agilent-6540 UHD accurate mass LC-QTOF-MS equipped with an electrospray ionization (ESI) interface (Agilent Technologies, Singapore). HPLC analysis was performed using the Prominence HPLC chromatograph system (Shimadzu, Kyoto, Japan). The chromatograph system was comprised of a binary pump system (LC-20AT) coupled with a UV/VIS detector (SPD-20A), communicator (CBM-20A), and degasser (DGU-20A_3_). HPLC analysis was conducted on a Phenomenex Luna^®^ C18 column (150 mm × 4.6 mm, 5 μm) (Phenomenex, Torrance, CA, USA).

### 4.2. Plant Extraction

#### 4.2.1. Crude Hydro-Ethanolic Extract

The fresh leaves of *D. cochinchinensis* were thoroughly cleaned and rinsed with water, then cut into small pieces and dried in a hot-air oven (Memmert UF 75, Schwabach, Germany) at 60 °C for 3 days. Subsequently, the dried leaves were ground into a rough powder using a grinder. The particle size of the powder was controlled by passing it through 100 mesh sieves (Retsch GmbH, Haan, Germany). The powdered leaves of *D. cochinchinensis* were macerated with 95% ethanol (EtOH) under shaking for 3 days at room temperature. Afterwards, the plant residue was separated by filtration through a filter paper (Whatman International Ltd., Chalfont St. Giles, UK). The filtrate was then concentrated by evaporating the ethanol using a BÜCHI Rotavapor R-124 (BÜCHI Labortechnik AG, Flawil, Switzerland). The crude extract was stored at −20 °C to maintain its integrity and stability until further analysis.

#### 4.2.2. Liquid–Liquid Partition

The partitioning process was utilized to separate a group of compounds based on solvent polarity. The *D. cochinchinensis* leaf extract (25 g) was dissolved in 100 mL of methanol (MeOH) and sonicated for 30 min using an ultrasonic bath (Elmasonic S60H, Singen, Germany). It was then partitioned with hexane. The remaining MeOH fraction was adjusted with water to achieve a 20% MeOH solution, which was subsequently partitioned with ethyl acetate [8]. The resulting fractions were processed further: the hexane and EA fractions were concentrated to dryness at 40 °C using a rotary evaporator, while the 20% MeOH fraction was further dried using a freeze dryer (Thermo Electron Co., Ltd., Waltham, MA, USA) to remove the water. All extracts were stored in the dark at −20 °C until further analysis. Three fractions were obtained from the solvent partition: hexane (HE), 20% MeOH (WE), and ethyl acetate (EE).

### 4.3. Sample Analysis

#### 4.3.1. High-Performance Liquid Chromatography (HPLC)

The chemical profile of *D. cochinchinensis* leaf extract was investigated by preparing a solution at a concentration of 10 mg/mL, dissolved in 50% methanol. This solution, as well as extracts obtained from liquid–liquid partitioning (also prepared at 10 mg/mL and dissolved in methanol), was filtered through nylon syringe filters with a pore size of 0.45 µm before analysis. For HPLC analysis, the system conditions were set to match those used for the LC-MS/MS analysis.

#### 4.3.2. Liquid Chromatography Mass Spectrometry (LC-MS/MS)

For the at-line technique, the *D. cochinchinensis* leaf extract was prepared at a concentration of 20 mg/mL by dissolving it in 50% acetonitrile. The solution was then sonicated for 15 min and filtered through nylon syringe filters with a pore size of 0.45 µm.

For LC-MS/MS analysis of the ethyl acetate extract obtained from partitioning, the extract was prepared at a concentration of 10 mg/mL in 50% methanol. Chemical compounds were identified based on *m*/*z* data. Separations were performed using a Luna C18(2) column (4.6 mm × 150 mm, 5 μm particle size) from Phenomenex (Torrance, CA, USA), with the temperature maintained at 35 °C. The mobile phase consisted of 0.1% formic acid in water (A) and 0.1% formic acid in acetonitrile (B), with a gradient system increasing the proportion of solvent B from 5% to 95% over 25 min.

### 4.4. At-Line Technique

The at-line assay employing LC-MS/MS analysis utilized an Agilent 1260 Infinity Series HPLC system coupled to an Agilent 6540 UHD accurate mass QTOF-LC/MS, which was equipped with an electrospray interface (ESI). The leaf extract of *D. cochinchinensis* (20 μL of 20 mg/mL to 400 μg) was analyzed using the at-line technique with modifications based on the protocols outlined by Bhandari et al. [38]. The *D. cochinchinensis* leaf extract was injected into the LC-MS/MS system, and micro-fractions were collected in a 96-well plate. This procedure was repeated three times. The separations were conducted using a Luna C18(2) column (4.6 mm × 150 mm, 5 μm particle size) from Phenomenex (Torrance, CA, USA), with the temperature held constant at 35 °C. The mobile phase consisted of a mixture of 0.1% formic acid in water (A) and 0.1% formic acid in acetonitrile (B). The gradient program began with 10% solvent B, which was increased to 15% over 20 min, followed by a gradual increase to 30% over 25 min, 50% over 15 min, and 70% over 20 min. Finally, solvent B was ramped up to 100% over a total duration of 100 min. A post-time of 5 min was allowed for system re-equilibration before the next injection. The injection volume was set at 20 μL, and the flow rate was maintained at 0.5 mL/min. The eluent was split into two flows in 1:9 ratios. The majority of the eluent was collected in a 96-well plate micro-fractionation system with 30 s per well, while the minor portion was directed to an ESI-QTOF-MS system. The micro-fractions were dried using a sample concentrator (Techne, Cambridge, UK) and kept at −20 °C until test day. The ion source was operated in negative mode with the following conditions: drying gas flow of 10 L/min, gas temperature set to 350 °C, nebulizer pressure at 30 psig, capillary voltage of 3500 V, skimmer voltage set to 65 V, octapole RFV at 750 V, and fragmentor voltage at 250 V. All acquisition data were analyzed using MassHunter software (Agilent Technologies, Santa Clara, CA, USA). The MS and MS/MS fragmentation patterns of PDE5 inhibitor active micro-fractions were compared using a library search, including MassHunter Metlin metabolite PCD/PCDL (Agilent Technologies), the Human Metabolomics Database (https://www.hmdb.ca), ChemSpider (https://www.chemspider.com), and MassBank (https://www.massbank.eu). Molecular formulas were generated using Agilent MassHunter Qualitative Analysis Software B 06.0.

### 4.5. PDE5 Inhibitory Activity

#### 4.5.1. Sample Preparation for PDE5 Inhibitory Activity

The micro-fractions in the 96-well plate were dried using a sample concentrator. Afterward, the micro-fractions were dissolved in 25 μL of 5% DMSO for the assay, resulting in a final concentration of 1% DMSO.

#### 4.5.2. Enzyme Preparation

The human PDE5 plasmid, received from Professor Dr. Joseph A. Beavo of the University of Washington, Seattle, WA, USA, was sub-cloned into a pcDNA3 vector with an ampicillin-resistant gene. These plasmids were then scaled up and purified using the Invitrogen™ PureLink™ HiPure Plasmid Maxiprep Kit (Thermo Fisher Scientific, Waltham, MA, USA)). HEK293 cells were transfected with human PDE5 plasmid using Lipofectamine^®^ 2000 reagent as per the protocol. After 2 days of transfection, the cells underwent treatment with a selective antibiotic (G418, 1 mg/mL) for 7 days. Surviving cells were sub-cultured in DMEM supplemented with 10% FBS in 175 mm flasks until they reached 90–100% confluence. Cell harvesting was performed using a scraper and lysed with a sonicate probe in 1 mL of Tris buffer [50 mM of Tris pH 7.5, 2 mM of EDTA, 1 mM of DTT, and 1:100 of 100 mM of PMSF]. The homogenate was centrifuged at 14,000 rpm, 4 °C for 20 min, and the supernatant was utilized as a source of PDE5 enzyme.

#### 4.5.3. Experimental Protocols

The enzymatic assay consisted of a two-step process [13]. In the first step, the PDE5 enzyme was mixed with negatively charged [3H]-cGMP, either in the presence or absence of an inhibitor. The reaction mixture consisted of 25 μL of 10 mM EGTA, 25 μL of buffer C (containing 100 mM of Tris–HCl (pH 7.5), 100 mM of imidazole, 15 mM of MgCl_2_, and 1.0 mg/mL of BSA), and 25 μL of either, the extract or the solvent (5% DMSO), as a control. The substrate, 25 μL of [3H]-cGMP, was added to the reaction mixture and incubated at 30 °C for 10 min. After incubation, the reaction was stopped by placing the tube in boiling water for 1 min, followed by cooling for 5 min. In the second enzymatic reaction, 25 μL of snake venom containing 5′-nucleotidase were added to the mixture and incubated at 30 °C for 5 min to break the 5′ nucleotide bond of the product (negatively charged [3H] -5′GMP), releasing negatively charged phosphate and uncharged guanosine. Afterward, 250 μL of 20 mM of Tris–HCl (pH 6.8) were added to the reaction mixture, which was then applied to a QAE anion exchange resin column. The uncharged guanosine was eluted through the column and collected. These eluents were then mixed with Ultima Gold (scintillation cocktail), and the radioactivity was measured using a β counter (Tri-Crab^®^ 2910 TR) (Perkin Elmer, Inc., Waltham, MA, USA). Higher amounts of uncharged guanosine led to lower inhibition, and counter. The hydrolysis activity of the PDE5 enzymes was standardized to represent 20–30% of the total substrate counts. The percentage of hydrolysis and PDE5 inhibition were calculated using the following Equations (1) and (2).

(1) $${\%\;\mathrm{Hydrolysis}}_{\;\mathrm{sample}/\mathrm{control}}=\left[\frac{\;({\mathrm{CPM}\;}_{\mathrm{sample}/\mathrm{control}}-{\mathrm{CPM}\;}_{\mathrm{background}})}{\left({\mathrm{CPM}\;}_{\mathrm{total}\;\mathrm{count}}-{\mathrm{CPM}}_{\;\mathrm{background}}\right)}\right]\;\times 100\;$$

CPM_sample_ is the radioactive count rate of the assay with an enzyme. CPM_background_ is the radioactive count rate of the assay, but without enzymes. CPM_control_ is the radioactive count rate of the assay with enzymes but without any sample. The CPM_total_ count is a count rate of 25 μL of substrate, plus 2 mL of low-salt buffer.

(2) $$\%\;\mathrm{PDE}5\;\mathrm{inhibition}\;=\left[1\;-\left(\frac{{\%\;\mathrm{Hydrolysis}\;}_{\mathrm{sample}}}{{\%\;\mathrm{Hydrolysis}\;}_{\mathrm{control}}}\right)\right]\;\times 100\;$$

The percentage of hydrolysis_sample_ and percentage of hydrolysis_control_ are the enzyme activities of the sample and solvent in the assay, respectively.

## 5. Conclusions

This is the first report demonstrating PDE5 inhibitory activity in *D. cochinchinensis* leaf extract, a high-value plant native to Asia. This finding introduces a novel biological activity for this species. Additionally, the study identifies the chemical constituents in the leaf that have the potential to act as PDE5 inhibitors. The at-line technique was employed to rapidly determine the chemical composition and assess the biological activity. Our study was able to isolate specific compounds in the bioactive region from partitioning, identifying them as potential candidates for PDE5 inhibitors in this plant. It would be highly beneficial to conduct further studies in the future on the aspects of compound purification, standardization of extract, stability study, and cytotoxicity testing to enhance the completeness of the study. Based on the overall conclusions, this study identified a novel natural PDE5 inhibitor in the leaf extract of *D. cochinchinensis*. Future research will focus on isolating the active compounds based on the data obtained in this study, determining their IC_50_ values, elucidating their chemical structures, and standardizing the extract. Additionally, comprehensive efficacy and safety evaluations will be conducted to support the development of this extract in addressing male erectile dysfunction.

## Figures and Tables

**Table 1:** PDE5 inhibition and IC_50_ value of five parts of *D. cochinchinensis* extract. The values represent the means ± standard deviations from triplicate experiments.

Part of Plant	% PDE5 Inhibition at 25 µg/mL	IC_50_ Values (μg/mL)
Leaf	100.00 ± 0.00	1.53 ± 0.12
Twig	97.57 ± 7.31	3.37 ± 0.54
Fruit	89.64 ± 2.69	14.92 ± 2.85
Heartwood	86.50 ± 0.93	19.05 ± 5.60
Bark	70.77 ± 4.23	16.03 ± 2.92

**Table 2:** Tentative identification by MS/MS fragmentation (negative mode) of *D. cochinchinensis* Leaf ethanolic extract.

Compound	Retention Time	*m*/*z*	Molecular Weight	Adduct	MS/MS Fragmentation	Tentative Identification	Formula	Error (ppm)	Classification
1	3.314	473.1573	474.1585	[M − H]^−^	341.1047, 179.0521, 131.1465, 118.0478, 101.0211, 89.0215	D-Galactopyranosyl-(1→3)-D-galactopyranosyl-(1→3)-L-arabinose	C_17_H_30_O_15_	−12.91	Oligosaccharide
2	3.504	165.0378	120.0423	[M + Cl]^−^	129.0156, 117.0160, 105.0165, 87.0067	2, 3-Dihydroxybutanoic acid	C_4_H_8_O_4_	16.13	Dihydroxybutyric acid
3	3.561	191.0548	192.0634	[M − H]^−^	173.0423, 131.0311, 120.0613, 111.0425, 87.0063	Quinic acid	C_7_H_12_O_6_	6.87	*Cyclohexanecarboxylic acid*
4	3.626	207.0492	208.0583	[M − H]^−^	173.0419, 155.0311, 129.0155, 117.0166, 93.0319	4-*O*-Methylglucuronic acid	C_7_H_12_O_7_	8.82	Glucuronic acid
5	3.762	173.0428	174.0528	[M − H]^−^	93.0325, 83.0471, 73.0275, 65.0360, 59.0124	Shikimic acid	C_7_H_10_O_5_	15.87	Phenolic cyclohexanecarboxylic acid
6	4.495	451.2173	452.2258	[M − H]^−^	419.1175, 305.1151, 179.0514, 125.0215, 96.9565	Calaliukiuenoside	C_20_H_36_O_11_	2.63	*Fatty acyl glycoside*
7	10.813	255.0470	256.0583	[M − H]^−^	193.0483, 123.0411	(2*R*, 3*S*)-Piscidic acid	C_11_H_12_O_7_	15.79	Phenylpropanoic acid
8	13.239	337.0870	338.1002	[M − H]^−^	239.0499, 191.0545, 163.0365, 119.0473	3-*O*-p-Coumaroylquinic acid	C_16_H_18_O_8_	17.48	Phenolic acid
9	13.645	239.0525	240.0634	[M − H]^−^	179.0344, 107.0494, 59.0135	3-(6-hydroxy-7-methoxy-2H-1,3-benzodioxol-5-yl)propanoic acid	C_11_H_12_O_6_	15.11	Benzodioxole
10	16.270	457.1297	458.1424	[M − H]^−^	163.0366, 119.0473, 101.0204, 89.0213, 71.0108, 59.0121	*cis*-p-Coumaric acid 4-[apiosyl-(1→2)-glucoside]	C_20_H_26_O_12_	11.92	Phenolic glycoside
11	17.550	577.2407	578.2516	[M − H]^−^	533.2540, 325.1095, 205.0672, 163.0576, 119.0320	Orbiculin A	C_33_H_38_O_9_	6.25	Sesquiterpenoid
12	17.754	439.1755	394.1780	[M + HCOO]^−^	393.1706, 265.0981, 205.0722, 163.0586, 119.0377	Cudraxanthone G	C_24_H_26_O_5_	1.65	Xanthone
13	20.275	755.1916	756.2113	[M − H]^−^	517.0541, 457.0353, 300.0268	Quercetin 3-rhamnosyl-(1→4)-rhamnoside-7-galactoside	C_33_H_40_O_20_	16.44	Flavonol glycoside
14	21.180	561.1312	562.1475	[M − H]^−^	451.0945, 391.0736, 289.0677, 257.0345, 161.0200, 137.0205, 125.0213	Epifisetinidol-(4β→8)-catechin	C_30_H_26_O_11_	16.10	Proanthocyanidin
15	22.581	340.1099	295.1056	[M + HCOO]^−^	188.0570, 161.0460	Prulaurasin	C_14_H_17_NO_6_	−17.96	Cyanogenic glycoside
16	28.788	431.1982	386.1941	[M + HCOO]^−^	385.1906, 179.0573, 89.0246	Citroside A	C_19_H_30_O_8_	−13.75	Terpene glycoside
17	29.630	475.1888	430.1839	[M + HCOO]^−^	429.1793, 265.0934, 205.0729, 163.0616, 101.0245	Phenethyl rutinoside	C_20_H_30_O_10_	−14.10	*O*-glycoside
18	30.100	739.2191	740.2164	[M − H]^−^	575.1405, 393.0638, 284.0336, 227.0348, 151.0029	Kaempferol 3-(2″-rhamnosylrutinoside) or Clitorin	C_33_H_40_O_19_	−13.52	*Flavonol glycoside*
19	31.800	609.1351	610.1534	[M − H]^−^	300.0276	Rutin	C_27_H_30_O_16_	18.07	*Flavonol glycoside*
20	31.904	523.2307	478.2355	[M + HCOO]^−^	477.2271, 161.0415, 113.0197, 101.0213	Cowanin	C_29_H_34_O_6_	5.81	Xanthone
21	31.911	725.2018	726.2007	[M − H]^−^	284.0278	Kaempferol 7-methyl ether 3-apiosyl-(1→5)-apioside-4′-glucoside	C_32_H_38_O_19_	−11.51	Flavonol glycoside
22	34.295	223.0569	224.0685	[M − H]^−^	163.0419, 91.0554	Sinapic acid	C_11_H_12_O_5_	19.26	Hydroxycinnamic acid
23	36.233	545.1496	546.1526	[M − H]^−^	435.0995, 409.0886, 393.0870, 289.0677, 255.0626	Guibourtinidol-(4α→6)-catechin	C_30_H_26_O_10_	−7.85	Proanthocyanidin
24	37.934	144.0459	145.0528	[M − H]^−^	126.0304, 115.0395, 65.9985	4-Hydroxyquinoline	C_9_H_7_NO	−2.86	Quinoline derivative
25	38.337	859.2154	814.2168	[M − HCOO]^−^	300.0216, 284.0271, 255.0213	Kaempferol 3-glucosyl-(1→2)-(6″-acetylgalactoside)-7-glucoside	C_35_H_42_O_22_	−0.49	Flavonol glycoside
26	38.445	901.2252	902.2481	[M − H]^−^	755.1893, 300.0220	Quercetin 3-(4″-(*E*)-p-coumarylrobinobioside)-7-rhamnoside	C_42_H_46_O_22_	17.31	Flavonol glycoside
27	38.680	833.1956	834.216	[M − H]^−^	790.5633, 681.1499, 600.1189, 561.1297, 451.0993, 271.0559, 161.0201	Epifisetinidol-(4β→8)-epicatechin-(6→4β)-epifisetinidol	C_45_H_38_O_16_	15.73	Proanthocyanidin
28	39.425	415.2003	370.1992	[M + HCOO]^−^	255.0996, 179.0524, 119.0309	5-Megastigmen-7-yne-3, 9-diol 9-glucoside	C_19_H_30_O_7_	−7.09	Fatty acyl glycoside
29	40.553	551.1661	552.1843	[M − H]^−^	429.0944, 293.0851, 257.0780, 152.0079, 135.0418	(*Z*)-Resveratrol 3, 4′-diglucoside	C_26_H_32_O_13_	19.80	Stilbene glycoside
30	41.813	871.2164	872.2223	[M − H]^−^	725.1816, 284.0287	Kaempferol 3-neohesperidoside-7-(6″-malonylglucoside)	C_37_H_44_O_24_	−1.63	Flavonol glycoside
31	41.961	885.2321	886.2532	[M − H]^−^	739.2104, 284.0341, 227.0326, 145.0298	Kaempferol 3-(4″-(*E*)-p-coumarylrobinobioside)-7-rhamnoside	C_42_H_46_O_21_	15.57	Flavonol glycoside
32	42.817	419.1375	420.1420	[M − H]^−^	257.0776, 135.0418, 121.0268, 109.0263	2′, 4′, 6′-Trihydroxydihydrochalcone 2′-glucoside	C_21_H_24_O_9_	−6.55	Chalcone glycoside
33	42.962	915.2427	916.2137	[M − H]^−^	817.2007, 739.1996, 561.1319, 284.0287, 255.0608	Kaempferol 3-ferulylrobinobioside-7-rhamnoside	C_43_H_48_O_22_	15.02	Flavonol glycoside
34	43.108	181.0516	182.0579	[M − H]^−^	153.0165, 108.0184	Ethyl 3,4-dihydroxybenzoate	C_9_H_10_O_4_	−5.34	Hydroxybenzoic acid ester
35	44.364	278.1052	279.1107	[M − H]^−^	188.0680, 172.0338, 144.0784, 114.0166, 96.9902	Unidentified	C_14_H_17_NO_5_	−6.49	-
36	44.645	843.2213	798.2219	[M + HCOO]^−^	284.0348, 227.0364	Kaempferol 3-*O*-[2″-(4″′-acetyl-rhamnosyl)-6″-glucosyl] glucoside	C_35_H_42_O_21_	−1.47	Flavonol glycoside
37	52.228	521.2244	522.2254	[M − H]^−^	475.2111, 307.0991, 247.0750, 205.0662, 167.1043, 145.0445, 113.0215	Carpelastofuran	C_30_H_34_O_8_	−12.10	Oxepine-fused flavone
38	55.313	193.0512	194.0579	[M − H]^−^	92.0244, 78.9563	Unidentified	C_10_H_10_O_4_	−2.94	-
39	56.454	327.2124	328.2250	[M − H]^−^	309.2005, 291.1921, 39.1224, 183.1331, 155.1031	9*S*, 12*R*, 13*S*-trihydroxy-10*E*, 15*Z*-octadecadienoic acid	C_18_H_32_O_5_	16.19	Long-chain fatty acid
40	58.836	329.2288	330.2406	[M − H]^−^	171.0985, 139.1072	12, 13, 15-trihydroxy-9*E*-octadecenoic acid	C_18_H_34_O_5_	13.81	Long-chain fatty acid
41	59.895	444.1316	399.1352	[M + HCOO]^−^	265.0683, 135.0401, 121.0262, 110.0223, 77.0376	Niaziminin	C_18_H_25_NO_7_S	4.00	Flavone *C*-glycoside
42	60.545	255.0669	256.0736	[M − H]^−^	109.0295	Liquiritigenin	C_15_H_12_O_4_	−2.42	Flavanone
43	61.468	267.0670	268.0736	[M − H]^−^	252.0384, 223.0353, 195.0394, 135.0056	Dalbergin	C_16_H_12_O_4_	−2.69	Neoflavone
44	62.079	753.2303	754.2320	[M − H]^−^	513.1424, 239.0706, 125.0196	2″′-*O*-Rhamnosyl-2″-*O*-glucosylcytisoside	C_34_H_42_O_19_	−7.36	Flavone *C*-glycoside
45	63.155	993.2959	754.2320	[M − H]^−^	643.1819, 513.1440, 137.0244	Swertisin 4′-*O*-glucoside-2″-*O*-rhamnoside	C_34_H_42_O_19_	−8.03	Flavone *C*-glycoside
46	64.993	601.1989	556.2075	[M + HCOO]^−^	555.1591, 445.1207, 419.1073, 255.0621, 135.0419, 109.0273	Punaglandin 1	C_27_H_37_ClO_10_	11.36	Prostanoid
47	67.996	895.4942	850.5079	[M + HCOO]^−^	509.4032, 439.3613, 205.0738	Hebevinoside XI	C_46_H_74_O_14_	13.24	Cucurbitacin glycoside
48	69.308	711.3261	676.3670	[M + Cl]^−^	675.3562, 397.1287, 277.2118	Gingerglycolipid A	C_33_H_56_O_14_	14.49	Glycosylmonoacylglycerol
49	73.818	339.1946	304.2250	[M + Cl]^−^	206.0616, 183.0087, 78.9588	11, 12, 15-trihydroxy palmitic acid	C_16_H_32_O_5_	−0.66	Long-chain fatty acid

Note: Mass errors between observed and theoretical *m*/*z* values are reported in parts per million (ppm). A tolerance of up to ±20 ppm was accepted for tentative identification due to sample complexity and instrumental limitations [35].

**Table 3:** Reviews of phytochemicals in the leaves of various species of genus *Dalbergia* [19,24].

Compound Isolated from Leaf	Scientific Name	Bioactivity	Classification
Alpinetin	*D. sissoo*	Anti-inflammatory, Anti-Osteoporosis, cytotoxic	Flavanone
β-Amyrin	*D. sissoo*		Triterpinoid
Biochanin A	*D. sissoo*		Isoflavone
Biochanin A 7-*O*-[β-D-apiofuranosyl-(1→5) -β-D-apiofuranosyl-(1→6)-β-D glucopyranoside]	*D. sissoo*		Isoflavone
Biochanin A 7-*O*-apiosyl-(1→6)-glucoside	*D. sissoo*		Isoflavone
Biochanin A 7-*O*-glucoside	*D. sissoo*	Anti-Osteoporosis	Isoflavone
Caviunin 7-*O*-β-D-glucopyranoside	*D. sissoo*		Isoflavone
Caviunin 7-*O*-[β-D-apiofuranosyl-(1→6)-β-D glucopyranoside]	*D. sissoo*	Anti-Osteoporosis	Isoflavone
Cavunin	*D. paniculata*		Isoflavone
Coromandelin	*D. coromandeliana*		Isoflavone
Dalsissooside (Caviunin 7-*O*-[β-D-apiofuranosyl-(1→6)-β-D-glucopyranoside])	*D. sissoo*	Anti-Osteoporosis	Isoflavone
Genstein	*D. sissoo*	Anti-Osteoporosis, cytotoxic	Isoflavone
Genistein 8-*C*-glucoside	*D. sissoo*		Isoflavone
5-Hydroxy-6,7-dimethoxy-4′-*O*-(6-*O*-D-apio-β-D-furanosyl-β-D-glucopyranosyl) isoflavone (7-Methyltectorigenin 4′-*O*-[β-D-apiofuranosyl-(1→6)-β-D-glucopyranoside])	*D. nigra*		Isoflavone
Kaemferol-3-*O*-β-D-glucopyranoside	*D. sissoo*		Flavone
Kaemferol-3-*O*-rutinoside	*D. sissoo*		Flavone
Norartocarpetin	*D. sissoo*		Flavone
Luteolin	*D. stipulacea*		Flavone
Luteolin 4′-rutinoside	*D. spruceana*		Flavone
7-Methyl tectorigenin 4′-*O*-[β-Dapiofuranosyl-(1→6)-β-D-glucopyranoside]	*D. sissoo*		Flavone
Muningin	*D. sissoo*		Isoflavone
7-*O*-Methyl tectorigenin 4′-*O*-galactoside	*D. spinosa*	Anti-Osteoporosis	Flavone
Pratensein	*D. sissoo*	Anti-Osteoporosis	Isoflavone
Prunasin	*D. spinosa*		Cyanogenic glycosides
Prunetin	*D. sissoo*		Isoflavone
Prunetin 4′-*O*-[β-D-apiofuranosyl-(1→6)-β-D glucopyranoside]	*D. sissoo*		Isoflavone
Prunetin 4′-*O*-galactoside	*D. spinosa*		Isoflavone
Quercetin 3-β-D-glucopyranoside	*D. sissoo*		Flavone
Quercetin-3-*O*-rutinoside	*D. sissoo*		Flavone
β-Sitosterol	*D. sissoo*		Phytosterol
Sissotrin	*D. sissoo*		Isoflavone
Sissooic acid	*D. sissoo*		Simple phenol
Stigmasterol	*D. sissoo*		Phytosterol
Syringaresinol-4″-*O*-β-D-monoglucoside	*D. sissoo*		Lignan
Tectorigenin	*D. sissoo*		Isoflavone
Tectorigenin 7-*O*-[β-D-apiofuranosyl-(1→6)-β-D-glucopyranoside]	*D. sissoo*		Isoflavone

**Table 4:** Tentative identification by MS/MS fragmentation (negative mode) of EE partition from *D. cochinchinensis*.

Compound	Retention Time	*m*/*z*	Molecular Weight	Adduct	MS/MS Fragmentation	Tentative Identification	Formula	Error (ppm)	Classification
1	5.555	169.0135	170.0208	[M − H]^−^	125.0241	Gallic acid	C_7_H_6_O_5_	4.42	Phenolic acid
2	7.228	313.0903	314.0975	[M − H]^−^	269.1021, 161.0449, 101.0248	(*S*)-Mandelic acid *O*-β-D-Glucopyranoside	C_14_H_18_O_8_	8.28	*O*-glycoside
3	7.488	153.0190	154.0262	[M − H]^−^	109.0296	Protocatechuic acid	C_7_H_6_O_4_	2.17	Phenolic acid
4	8.301	239.0549	240.0621	[M − H]^−^	179.0346, 149.0606, 107.0502	Eucomic acid	C_11_H_12_O_6_	5.07	Phenolic acid
5	8.337	435.1257	436.1369	[M − H]^−^	273.0756, 123.0439	Phloretin-4′-*O*-glucoside	C_21_H_24_O_10_	9.13	Dihydrochalcone glucoside
6	8.947	137.0243	138.0316	[M − H]^−^	108.0218	Protocatechuic aldehyde	C_7_H_6_O_3_	0.86	Phenolic aldehyde
7	8.993	739.2000	740.2070	[M − H]^−^	575.1345, 284.0317	Kaempferol 3-rhamnosyl-(1→3)-rhamnosyl-(1→6)-glucoside	C_33_H_40_O_19_	12.31	Flavonol glycoside
8	9.173	561.1336	562.1407	[M − H]^−^	409.0905, 289.0707, 245.0811, 161.0240, 109.0294	Catechin-Afzelechin	C_30_H_26_O_11_	11.82	Procyanidin
9	9.374	475.1777	430.1839	[M + HCOO]^−^	429.1736, 265.0921, 205.0704, 163.0602	Phenethyl rutinoside	C_20_H_30_O_10_	9.26	*O*-glycosyl
10	9.541	340.1008	295.1026	[M + HCOO]^−^	188.0559, 161.0453	Prulaurasin	C_14_H_17_NO_6_	8.79	Cyanogenic glycoside
11	9.989	593.1436	594.1509	[M − H]^−^	284.0316	Kaempferol 3-glucoside-7-rhamnoside	C_27_H_30_O_15_	12.80	Flavonol glycoside
12	10.364	859.2178	814.2168	[M + HCOO]^−^	284.0307	Kaempferol 3-glucosyl-(1→2)-(6″-acetylgalactoside)-7-glucoside	C_35_H_42_O_22_	−3.29	Flavonol glycoside
13	10.377	901.2282	902.2354	[M − H]^−^	755.1964, 300.0257	Quercetin 3-(4″-(*E*)-p-coumarylrobinobioside)-7-rhamnoside	C_42_H_46_O_22_	13.98	Flavonol glycoside
14	10.653	545.1396	546.1467	[M − H]^−^	409.0903, 289.0705, 255.0655, 205.0488	Afzelechin dimer	C_30_H_26_O_10_	10.49	Procyanidin
15	10.914	833.1965	834.2038	[M − H]^−^	681.1548, 561.1345, 529.1093, 289.0700, 161.0244	Epifisetinidol(4β→8) epicatechin(6→4β)epifisetinidol	C_45_H_38_O_16_	14.65	Procyanidin
16	10.982	915.2437	916.2508	[M − H]^−^	739.2026, 284.0314	Kaempferol 3-ferulylrobinobioside-7-rhamnoside	C_43_H_48_O_22_	13.93	Flavonol glycoside
17	11.300	844.2297	784.2062	[M + CH_3_COO]^−^	284.0319	Kaempferol 3-*O*-(2″-rhamnosyl-6″-acetyl-galactoside) 7-*O*-rhamnoside	C_34_H_40_O_21_	−2.15	Flavonol glycoside
18	12.043	817.2030	818.2102	[M − H]^−^	665.1599, 561.1360, 409.0903, 289.0707, 255.0653, 161.0235	Afzelechin trimer	C_45_H_38_O_15_	15.53	Procyanidin
19	12.702	801.2091	802.2262	[M − H]^−^	665.1601, 545.1412, 409.0901, 289.0706, 255.0654	Afzelechin trimer	C_45_H_38_O_14_	12.21	Procyanidin
20	13.055	529.1440	530.1512	[M − H]^−^	393.0965, 255.0657, 137.0242	Afzelechin Guibourtinidol	C_30_H_26_O_9_	12.11	Procyanidin
21	13.928	785.2180	786.2312	[M − H]^−^	649.1654, 529.1460, 393.0956, 289.0700, 255.0653	Afzelechin Guibourtinidol dimer	C_45_H_38_O_13_	7.60	Procyanidin
22	15.912	444.1254	399.1318	[M + HCOO]^−^	398.1207, 292.0799, 121.0234	Adlumidiceine	C_21_H_21_NO_7_	10.37	Alkaloid

Note: Mass errors between observed and theoretical *m*/*z* values are reported in parts per million (ppm). A tolerance of up to ±20 ppm was accepted for tentative identification due to sample complexity and instrumental limitations [35].

## Data Availability

The original contributions presented in this study are included in the article. Further inquiries can be directed to the corresponding author.

## Abbreviations

The following abbreviations are used in this manuscript:

PDE5	Phosphodiesterase 5
HE	Hexane-partition extract
EE	Ethyl acetate-partition extract
WE	Water-partition extract
